# Radiogenomic Analysis of F-18-Fluorodeoxyglucose Positron Emission Tomography and Gene Expression Data Elucidates the Epidemiological Complexity of Colorectal Cancer Landscape

**DOI:** 10.1016/j.csbj.2019.01.007

**Published:** 2019-01-25

**Authors:** Efstathios–Iason Vlachavas, Eleftherios Pilalis, Olga Papadodima, Dirk Koczan, Stefan Willis, Sven Klippel, Caixia Cheng, Leyun Pan, Christos Sachpekidis, Alexandros Pintzas, Vasilis Gregoriou, Antonia Dimitrakopoulou-Strauss, Aristotelis Chatziioannou

**Affiliations:** a Institute of Biology, Medicinal Chemistry & Biotechnology, National Hellenic Research Foundation, Athens, Greece; b Department of Molecular Biology and Genetics, Democritus University of Thrace, 68100 Dragana, Greece; c Enios Applications Private Limited Company, A17671 Athens, Greece; d Core Facility Micro-Array-Technology, Center of Medical Research, University of Rostock, Germany; e Surgical Clinic A, Klinikum Ludwigshafen, Germany; f Clinical Cooperation Unit Nuclear Medicine, German Cancer Research Center, Heidelberg, Germany

**Keywords:** ^18^F-FDG PET, Radiogenomics, Colorectal cancer, Microarray analysis, Translational bioinformatics, TCGA, AUC, Area Under the Curve, ACADM, Acyl-Coenzyme A Dehydrogenase, CRC, Colorectal cancer, CCT7, Chaperonin Containing TCP1 Subunit 7, CD44, CD44 Molecule (Indian Blood Group), DE, Differentially Expressed, FD, Fractal Dimension, FDG, F-18-Fluorodeoxyglucose, GDC, Genomics Data Commons, GEO, Gene Expression Omnibus, GSTP1, Glutathione S-Transferase Pi 1, KIT, Proto-Oncogene Receptor Tyrosine Kinase, Lasso, least absolute shrinkage and selection operator, MFA, Multiple Factor Analysis, PET, Positron Emission Tomography, PCs, Principal Components, ROC, Receiver-operator Characteristic curve, SUV, Standardized Uptake Value, TCGA-COAD, The Cancer Genome Atlas-Colon Adenocarcinoma

## Abstract

**Purpose:**

Transcriptomic profiling has enabled the neater genomic characterization of several cancers, among them colorectal cancer (CRC), through the derivation of genes with enhanced causal role and informative gene sets. However, the identification of small-sized gene signatures, which can serve as potential biomarkers in CRC, remains challenging, mainly due to the great genetic heterogeneity of the disease.

**Methods:**

We developed and exploited an analytical framework for the integrative analysis of CRC datasets, encompassing transcriptomic data and positron emission tomography (PET) measurements. Profiling data comprised two microarray datasets, pertaining biopsy specimen from 30 untreated patients with primary CRC, coupled by their F-18-Fluorodeoxyglucose (FDG) PET values, using tracer kinetic analysis measurements. The computational framework incorporates algorithms for semantic processing, multivariate analysis, data mining and dimensionality reduction.

**Results:**

Transcriptomic and PET data feature sets, were evaluated for their discrimination performance between primary colorectal adenocarcinomas and adjacent normal mucosa. A composite signature was derived, pertaining 12 features: 7 genes and 5 PET variables. This compact signature manifests superior performance in classification accuracy, through the integration of gene expression and PET data.

**Conclusions:**

This work represents an effort for the integrative, multilayered, signature-oriented analysis of CRC, in the context of radio-genomics, inferring a composite signature with promising results for patient stratification.

## Graphical Abstract

**Figure:**
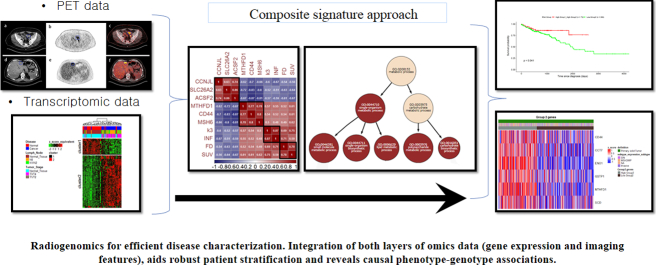

## 1 Introduction

The advent of “omics”-technologies (microarrays, and more recently the wave of Next Generation Sequencing (NGS) technologies) is revolutionizing oncological research and treatment, through the introduction of molecular signatures, as a key instrument for streamlining evaluation of disease onset and progression. Regarding colorectal cancer (CRC), the application of these high-throughput technologies has already highlighted significant genomic alterations and revealed specific genes with increased causality concerning pathological development [1,2].

However, the translation of the plethora of biomedical data derived from microarray experiments into clinical practice remains challenging, especially when the aim is the derivation of putative biomarkers. Colorectal tumors are extremely heterogeneous, involving distinct molecular pathways and epigenetic alterations [[3], [4], [5]]. Moreover, this complexity is further enhanced by the environmental crosstalk, as well as particularities of the isolation and collection of the biological material. This results in lack of reproducibility between different studies, routinely observed in various cases [6]. Thus, the pressure is intense for earlier, rapid, more accurate diagnostic, and ideally non-invasive therapeutic strategies. F-18-Fluorodeoxyglucose (FDG) is the most commonly utilized positron emission tomography (PET) radiopharmaceutical agent, which is transported and phosphorylated like glucose but then trapped in the cell. Although not routinely applied in initial CRC staging, FDG PET/CT represents an evolving modality with proven value in staging, detection of recurrence and therapy monitoring of colorectal tumors [7,8]. Here, we propose an analytical workflow for the integrative analysis of CRC data, through inference of robust, composite signatures encompassing both transcriptomics and PET measurements. The derived signatures capture key processes and pathways consistently engaged in CRC manifestation and were evaluated for their potential in tumor classification in an independent public RNA-seq dataset.

## 2 Methods

### 2.1 Patients, Tissue Specimens

A total of 30 patients (Table 1) with histologically confirmed, primary, untreated colorectal adenocarcinomas were studied. Among them, 13 patients were selected from a previous cohort [9], complemented by their gene expression measurements from both cancer and matched normal colon tissue. The tissue specimens of the tumor and normal colon were removed during surgery and immediately frozen in liquid nitrogen. Total RNA was extracted for further processing and microarray hybridization, as previously described [9] (with the difference that Affymetrix HG-U133plus2 platform was used). The quality of isolated RNA was evaluated photometrically using the 260/280 ratio and on an agarose gel. All studies have been approved by the Ethical Committee of the University of Heidelberg and the Federal Agency for Radiation Protection. Detailed information for each patient, is included in the Supplementary Table S1.

**Table 1:** Basic clinicopathological characteristics of the 30 total patients in the two microarray datasets. Overall, the patients represent a relatively homogenous cohort with only primary colorectal cancer adenocarcinomas.

Patient Characteristics		Microarray Platform	
		hgu133a (13 patients)	hgu133plus2 (17 patients)
		Number of patients	
Sex	F	7	8
	M	6	9
Tumor Stage	T1	1	1
	T2	–	6
	T3	10	10
	T4	2	–
Lymph Node Status	N0	7	12
	N1	2	1
	N2	4	4
Synchronous distinct metastasis (M)		4	2
Anatomic Location	Right-sided	7	8
	Left-sided	6	9
Mean Age, (range)		70 (58–81)	64 (51–83)

### 2.2 FGD PET Data and Procedures

**Fig. 1:**
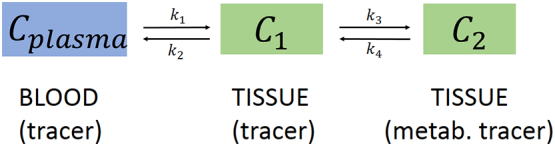
Schematic presentation of the two-tissue compartment. C*plasma* represents the tracer concentration in blood, C1 the unbound (non-metabolized) tracer in tissue, and C2 the metabolized tracer in tissue. In case of FDG k1 is the transport rate of the tracer from blood to tissue, k2 the transport rate back to blood, k3 the phosphorylation rate and k4 the dephosphorylation rate.

The FDG dynamic PET (dPET) examinations were performed over the abdomen in all patients, using a 28-frame protocol for 60 min. Details regarding dPET data acquisition have already been described [10]. PET images have been iteratively reconstructed (6 iterations, 2 subsets) with the ordered subset expectation maximization (OSEM) algorithm. The dynamic evaluation of the PET data has been performed with the PMOD Software (PMOD Technologies LLC, Zuerich, Switzerland) (Supplementary Fig. S1). Volumes of Interest (VOIs) have been placed over the tumor area, the reference tissue (normal colon tissue) as well as in the descending aorta. Time activity curves have been calculated for all VOIs. Regarding data analysis, this was based on visual (qualitative) analysis of the FDG PET/CT scans, semi-quantitative evaluation based on standardized uptake value (SUV) calculations, and quantitative analysis based on a two-tissue compartment model. The two-tissue compartment model requires the use of an input function, which provides the tracer concentration in the vessels. We used an image-derived input function via a VOI placed over the descending aorta. The application of a two-tissue compartment model leads to the extraction of the kinetic parameters k1, k2, k3 and k4 as well as influx (INF-Ki), taking into account the fractional blood volume (VB). These indices describe specific molecular processes of FDG: k1 reflects the carrier-mediated transport of the tracer from plasma to tissue while k2 reflects the transport of FDG back from tissue to plasma, and k3 represents the phosphorylation rate while k4 the dephosphorylation rate of the radiotracer (Fig. 1). Influx (Ki) is derived from the equation = (k1 × k3)/(k2 + k3). In addition to performing compartment analysis, a non-compartment model based on the fractal dimension (FD) for the time-activity data was also applied. FD is a parameter of heterogeneity based on the box counting procedure of chaos theory [11].

### 2.3 Computational Methods

#### 2.3.1 Microarray Data Analysis

**Fig. 2:**
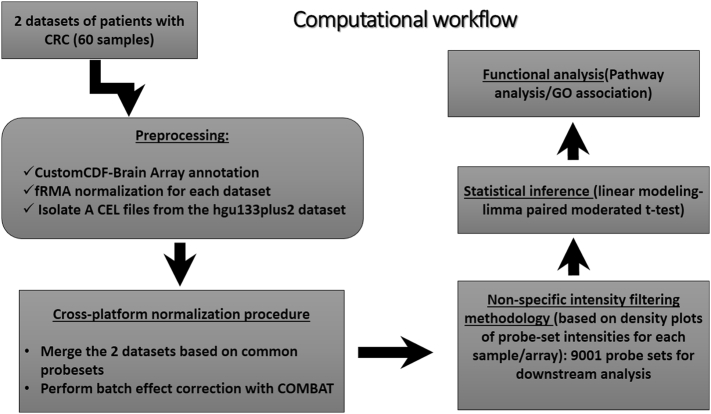
Computational workflow applied for the integrative analysis of the two microarray datasets. Complete analysis was performed in R statistical software/Bioconductor (R versions 3.2.2, 3.3.1 & 3.5.0). Details of the framework are described at the Supplementary Legend of Fig. 2.

Arrays HG-U133 and HG-U133plus2 were used for respectively 13 patients (data from previous study, GEO accession number GSE110225) and 17 patients from this study (data deposited in GEO, Accession Number GSE110225), in duplicates, comprising tumor and surrounding tissue (adjacent control). The two datasets were pre-processed, integrated and analyzed as a single dataset. Complete microarray analysis was performed with R (R versions 3.2.2, 3.3.1 and 3.5.0)/Bioconductor software [12], using custom CDF annotation [13] (Fig. 2). Technical details and statistical analyses for the derivation of differentially expressed genes are described at Supplementary Legend of Fig. 2.

#### 2.3.2 Functional Analysis of Gene Expression Data

In order to detect and rank significantly altered biological processes and their respective driver genes from the aforementioned statistical comparisons, we utilized the *BioInfoMiner* platform (https://bioinfominer.com) [14, 37], which performs pathway analysis, exploiting advanced statistical and network analysis criteria, applicable on various biological, hierarchical vocabularies (Gene Ontology, Reactome Pathways and MGI Mammalian Phenotype Ontology).

#### 2.3.3 Association of Gene Expression and Clinical Data

As the integrated dataset is highly heterogeneous, Spearman correlation analysis was used to produce rho Correlation coefficients and their relative asymptotic *P*-values (threshold: |cor| ≥ 0.6 & *p*-value <.05) in order to select the most correlated genes with any of the PET variables, from the initial list of the 911 DE common genes.

#### 2.3.4 Derivation and Evaluation of Informative Feature-Sets for CRC Classification

Gene features were selected from the subset of differentially expressed genes deriving from the microarray analysis using the limma R package [15], and prioritized by BioInfoMiner as master regulators. The data mining approach applied to the 3 different groups of features (only genes, only PET features and both types of data), is summarized in the following steps: (1) implementing a cross-validation scheme with a Random Forest classifier for each group separately (10 fold cross-validation, repeated 10 times for 10 different random seeds) (2) evaluation and comparison of the relative model performances by the aggregated resampling results (R package caret) [16]. Additionally, the contribution of each variable to the initial composite signature of the 102 features, was evaluated through Multiple Factor Analysis (R packages FactoMineR and FactoShiny) [17].

Finally, lasso feature selection was applied to derive the most important discriminative variables (genes and PET measurements), and the final feature subset was derived from the total aggregated results (R package glmnet: 100 different random seeds/10-fold cross validation, alpha = 1 and lambda = lambda.1se, for each random seed [18].

#### 2.3.5 Epidemiological Evaluation of the 22-Gene Signature in the TCGA COAD RNA-Seq Dataset

A dataset of 519 RNA-seq samples, from 41 solid tissue normal and 478 primary colon adenocarcinomas surgically removed from patients prior to therapeutic administration of chemotherapy or other therapies [19], was retrieved from the GDC (Genomic Data Commons) database, analyzed for gene expression and used as an independent dataset for evaluation of the classification capacity of the 22 gene signature [20], utilizing the R package TCGAbiolinks (Supplementary Fig. S2). The characterization and grouping of cancer samples based on the 22-gene signature, was performed as described below. Briefly, median expression value across all cancer samples is computed for each gene. For each sample a numeric value of 1 is assigned if its expression is higher than the median for each gene, or 0 if it is lower. For each gene-subgroup depicted in Fig. 5, an average expression score is computed for each sample. Finally, if the average expression score of a sample is >0.5, it is characterized as “Highly expressed” for the relative Group, else Low.

#### 2.3.6 Survival Analysis

Based on the pre-defined groups of patients described in the previous section, the prognosis of each cluster was examined by Kaplan-Meier overall survival estimators (R package TCGAbiolinks-TCGAanalyze_survival function). Moreover, the survival outcomes of the aforementioned groups were compared by log-rank tests (Surv function-R package survminer). Finally, the relative Hazard Ratios were computed with the R package survival (coxph function).

## 3 Results

### 3.1 Selection of Genes Characterizing Primary Colon Adenocarcinomas

**Fig. 3:**
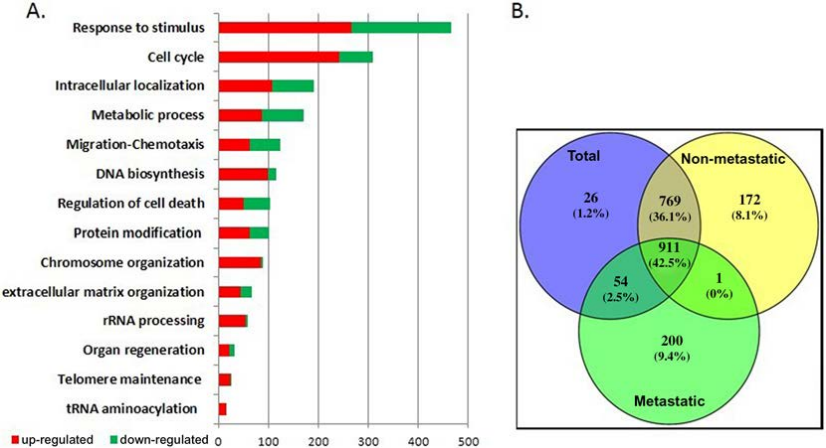
A. Top ranked GO Biological Processes resulting from the application of semantic analysis on the 1760 genes found as differentially expressed between cancer and normal tissue. GO terms identified as significantly enriched are grouped according to their biological relevance (horizontal axis). The vertical axis depicts the number of relevant genes. B. Common and unique DE genes found by the three statistical comparisons: all 30 cancer samples versus their paired controls (‘total comparison’), 24 cancer samples from patients without distant metastases versus their adjacent paired controls (‘non-metastatic’ comparison), and 6 cancer samples from patients with synchronous distant metastases versus their respective controls (‘metastatic’ comparison).

Differential expression analysis between tumor and adjacent tissue revealed a total of 1760 significantly Differentially Expressed (DE) genes, from which 916 were up-regulated, and 844 were down-regulated (Supplementary Table S2). Several cytokines were identified among the top-up-regulated genes (*CXCL3, CXCL1, CXCL2*), as also various cadherins (*CDH3*) and specific phosphoproteins (*SPP1*). On the other hand, the top down-regulated genes with the most significant decrease in expression, included *AQP8, CLCA4, MS4A12, CA1, GUCA2A, ZG16, SLC263, CA4, UGT2B17* & *SLC26A2*. The functional enrichment analysis of the 1760 DE genes, revealed key biological processes as significantly enriched, including among other, cellular response to stimulus, cell cycle, metabolism, chemotaxis and cell migration, DNA biosynthesis and regulation of cell death (Fig. 3). Interestingly, the majority of genes involved in cell cycle are up-regulated, including nodal genes like cyclin B1 (*CCNB1*) and cyclin dependent kinase (*CDK1*), both essential for mitosis progression, suggesting an uncontrolled cell proliferation. In addition, processes related to telomere maintenance, contain almost exclusively up-regulated genes (24 out of 25), supporting that this could enable replicative immortality, one of the important hallmarks of carcinogenesis [21]. The results of the functional analysis are shown in Fig. 3A.

For the derivation of a minimal gene set, characterizing cancer manifestation, two additional comparisons, one comprising 24 samples from patients without distant metastases and one with the remaining 6 patients with distant synchronous metastases, were performed (Supplementary Tables S3 & S4). The intersection of the 911 common genes, was retained as an initial consensus gene set characterizing primary colorectal adenocarcinomas (Fig. 3B). These genes were subjected to functional network analysis with BioInfoMiner web platform, implying the utilization of functional and pathway annotations as features driving the selection of important genes. This selection approach contributes to the generalization capability of the prioritized genes, because it preserves the underlying molecular basis. This analysis highlighted a subset of 94 linker genes (Supplementary Table S5), with pivotal role in the cross-talk among distinct molecular pathways, namely telomere length regulation, nuclear transport, growth, tumor suppression and structural abnormalities of the large intestine (abnormal crypts of Lieberkuhn morphology) (Supplementary Fig. S3).

### 3.2 Integration of Gene Expression and PET Variables

**Fig.4:**
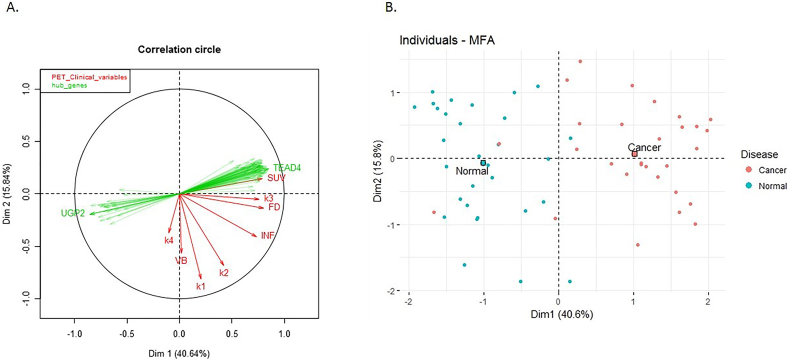
A. Correlation plot (FactoShiny R package) combining 94 selected linker genes (marked in green) with the 8 PET variables (marked in red). On the first PC (Dim1) genes significantly correlate with four of the PET variables (SUV, k3, FD, INF), whereas the 4 (k4, VB, k1, k2) are largely orthogonal (independent) and contribute additional information to the composite dataset (Dim2). B. Projection of the samples to the first two PCs, based on both PET and gene features (R package factoextra). First PC (Dim1), spanned by the 94 genes and 4 of the PET variables, clearly separates cancer samples from the adjacent control ones. (For interpretation of the references to colour in this figure legend, the reader is referred to the web version of this article.)

Integration of gene expression and PET data, resulted in a single composite feature-set, consisting of the 94 selected genes and the 8 PET variables. Multiple Factor Analysis (MFA) was performed, in order to correlate heterogeneous variables and measure their contributions to the total variation of the composite set. In Fig. 4 the results for the first 2 axes (Principal Components, PCs) are shown, where the simultaneous projection of both PET and gene expression data sets off interesting putative association patterns between the two data layers. In detail, in Fig. 4A we observe the high correlation of 4 PET variables (marked in red, SUV, k3, FD, INF) with a large group of genes. The correlation between the two (molecular and PET) variable groups is captured by the first PC (Dim1), which accounts for 40.64% of total variation. The second PC (Dim2), integrates the contributions of solely PET variables (k4, VB, k1 and k2). In the first dimension, genes and PET variables share equal contributions (PET = 49.98% & genes = 50.04%), while in the second dimension the PET variables dominate (~88% versus 12.14% attributed to genes). Hence, 4 PET variables out of 8 are correlated to gene expression, whereas the other 4 are largely orthogonal (independent) and contribute unique, additional variance, largely unaccounted by the molecular descriptors, to the composite dataset. In Fig. 4B, the separation of samples according to the same first 2 PCs, is shown.

This result supports the utilization of PET measurements for patient stratification, as on one hand, part of them correlates strongly with gene expression measures and can serve as proxies of the underlying molecular pathways and on the other hand, another part provide additional non-redundant information. This result also suggests a performance advantage of the composite feature-set, if both types of data are available.

### 3.3 Evaluation of the Feature-Sets through Classification Methods

**Fig. 5:**
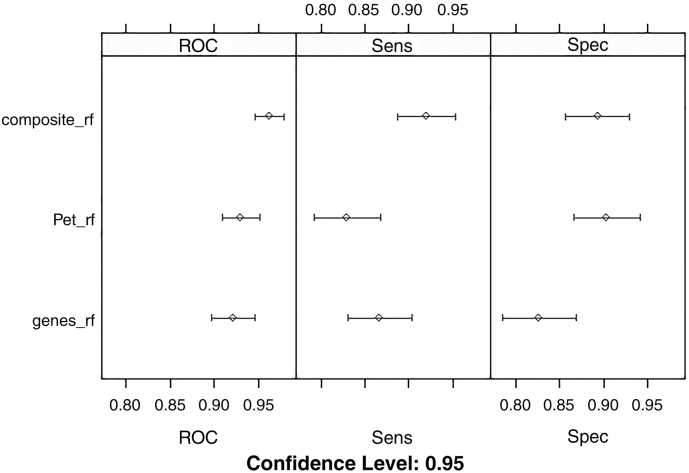
Grouped dotplots of the average cross-validation resampling results (representative example using a specific random seed), for the 3 groups of variables: only the 94 hub genes, only the 8 PET variables and the combination of both (ROC, Sensitivity, Specificity-10 fold cross-validation, repeated 10 times for 10 different random seeds). Using the total merged microarray dataset as the training set, the composite signature yields overall better performance measures than either PET, or genes separately.

The performances of the three feature-sets (Genes-only, PET-only & Composite) in regard to tumor classification were evaluated through the machine-learning, cross-validation scheme, with respect to the Sensitivity, Specificity and Receiver Operating Characteristic (ROC) metrics (Fig. 5). Whereas all perform well, the composite feature-set yields an overall better performance, showing the advantage of combining the gene expression data with the PET kinetic data. Notably ROC, which balances specificity and sensitivity, is significantly improved attaining an area under the curve (AUC) of 0.97. Furthermore, from the aggregated results of the cross validation procedure with Random Forests, the 94 genes showed an overall better sensitivity (that is predicting the cancer samples), whereas the 8 clinical quantitative variables illustrated a relatively higher specificity.

### 3.4 Derivation of a Composite Signature

**Fig. 6:**
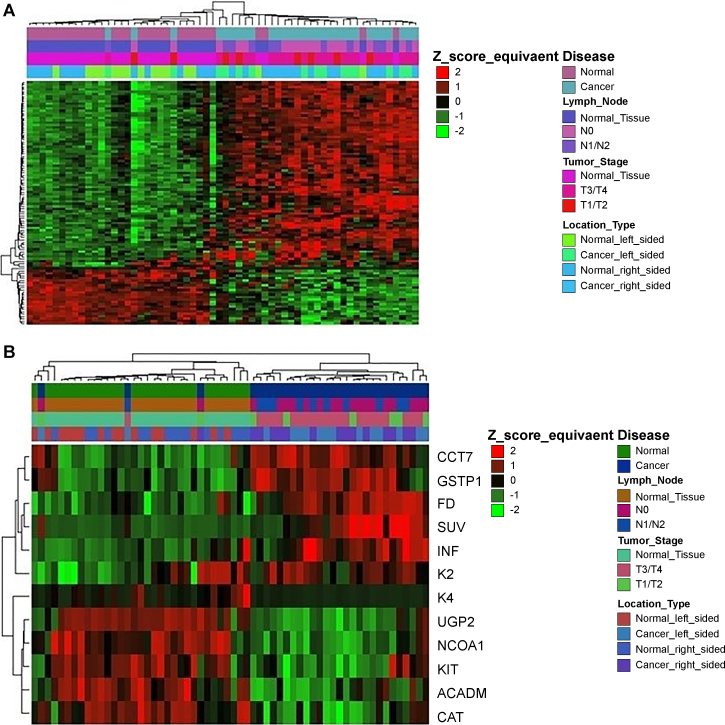
Comparative heatmap plots of hierarchical clustering (distance: Pearson, linkage: average, scaled log2 normalized intensity values) comparing different disease states of the microarray dataset, using the R package ComplexHeatmap [22] (distance: Pearson, linkage: average) A. Initial composite signature B. Lasso-optimized, compact composite signature of 7 genes and 5 PET variables. The compact composite signature preserves the discrimination potential of the initial signature.

The composite feature set (94 genes +8 PET variables) was further optimized by mathematical methods for dimensionality reduction, in order to derive the minimum set of features with maximum discrimination capability and thus to improve efficiency from the clinical point of view. On this purpose, lasso feature selection algorithm was applied (see Materials and Methods). This resulted to a compact composite signature that comprised 12 features, 7 genes and 5 PET variables, representing a reduced set of less covariant features, corresponding to discrete functional modules. In this sense, from the cluster of covariant PET variables, namely VB, k1, k2 and k4 (Fig. 4A), only k2 and k4 were selected as the best descriptors of the variance observed in Dim2. Moreover, it improved the separation of patients, leading to more homogeneous clusters and less mislabeled samples (Fig. 6).

### 3.5 Construction of an Expression Signature Comprising Genes as Proxies of PET Measurements

With the aim to elucidate the molecular mechanisms related to the PET variables, we performed correlation analysis between the initial list of 911 common DE genes and the 8 PET variables. The analysis pinpointed that 4 PET features, out of the 8, namely, INF FD, k3 and SUV had a significant correlation with 47 genes (Supplementary Table S6). In particular, SUV presented the highest number of correlations with the gene set, followed by k3, FD, and INF.

**Fig. 7:**
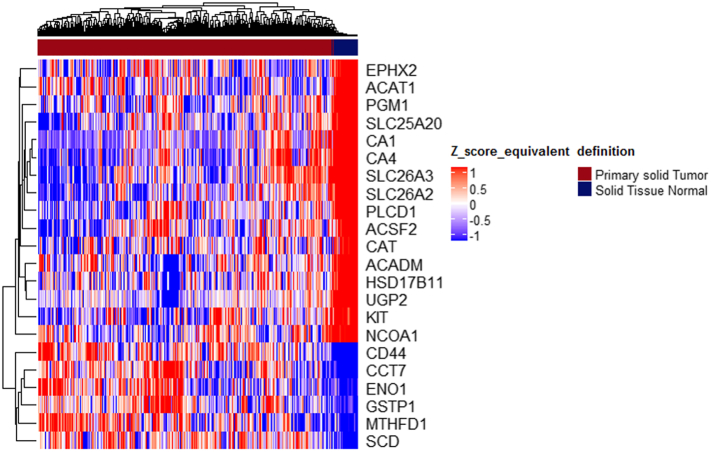
Heatmap of the 22-genes signature in the TCGA RNA-Seq dataset (456 cancer samples, 41 normal samples-R package ComplexHeatmap). Samples with relatively high expression of a given gene are marked in red and samples with relatively low expression are marked in blue. The gene set achieves an overall good separation of normal and cancer samples. Samples and genes have been reordered by the method of hierarchical clustering (average method, Pearson distance). (For interpretation of the references to colour in this figure legend, the reader is referred to the web version of this article.)

In contrast, k1, k2, k4 and VB did not show any significant correlations with any of the 911 genes, suggesting that these clinical variables, contribute important information, non-captured by the transcriptomic data. BioInfoMiner semantic analysis, using as input these 47 genes, was performed on 2 different controlled vocabularies, GO and Reactome, revealing various molecular mechanisms related to metabolism (one‑carbon metabolic process, ion transport, glycolytic process, carboxylic acid metabolic process, purine-containing compound metabolic process), as significantly enriched. Subsequent gene prioritization, resulted in 15 genes with putatively central role in the aforementioned pathways. Aiming to assess their potential clinical significance in the large TCGA cohort, we combined those 15 genes with the 7 genes of the compact composite signature. This 22-feature set, composed solely of gene expression values, was initially evaluated regarding its discriminatory potential in separating cancer from normal samples, utilizing the independent TCGA-COAD dataset, and illustrated a robust performance (Fig. 7).

**Fig. 8:**
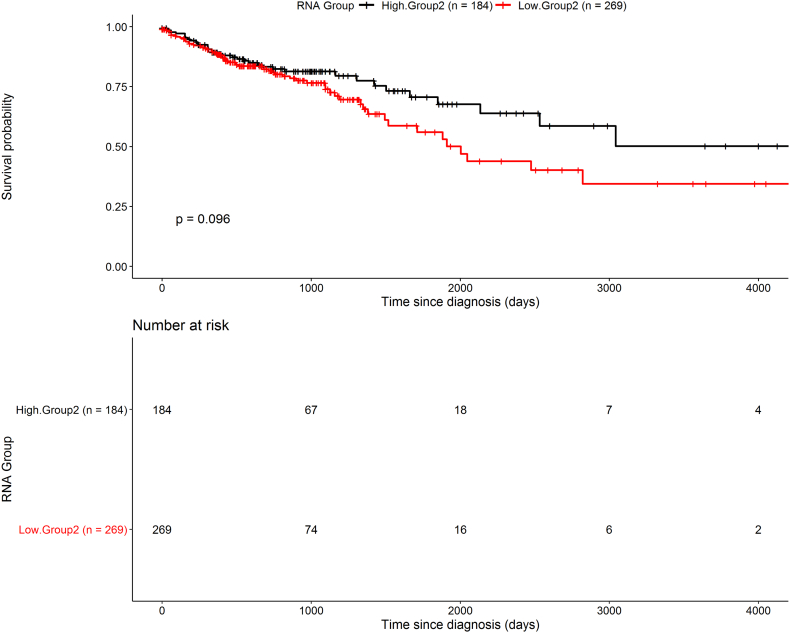
Kaplan-Meier plot of overall survival estimates, comparing the 2 patient clusters survival outcomes, based on the expression of the 6 genes of Group2 in the TCGA-COAD dataset (log-rank *p*-value-R package survmimer).

**Fig. 9:**
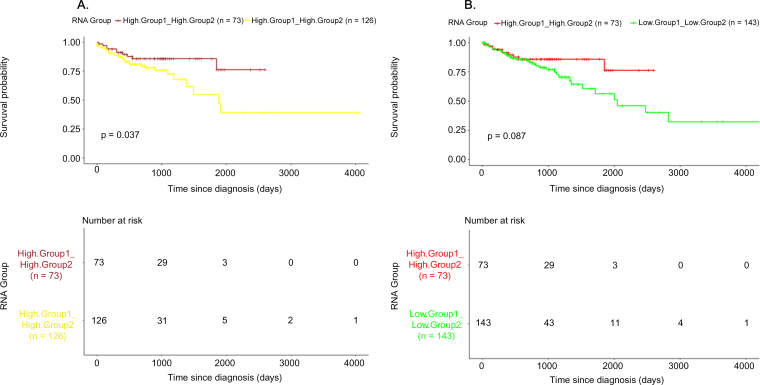
Kaplan-Meier plots of overall survival, examining the prognosis of the 4 final patient clusters using the 22- genes signature in the TCGA-COAD dataset, based only on the available cancer samples. Fig. 7A illustrates the overall survival estimates between the groups “HighGroup1.HighGroup2” and “HighGroup1.LowGroup2”, whereas Fig. 7B compares “HighGroup1.HighGroup2 and “LowGroup1.LowGroup2” (log-rank p-value-R package survminer).

The 22 genes were clearly separated into 2 distinct sub-groups, based on their expression pattern in the normal samples (Fig. 7). Specifically, Group1 contains 16 genes highly expressed in normal samples, while Group 2 contains 6 genes with low expression. Next, we ought to investigate if this small feature-set, could have any impact in patient stratification and potential prognostic significance. On this premise, we constructed a scoring methodology and characterized the cancer samples, based on the relative expression of the 2 separate sub-groups (see Materials and Methods). Hence, based on Group1, patients were classified either as “Group1 High” (high expression in the majority of the relevant genes) or “Group1 Low” (low expression in the majority of the relevant genes). Similarly, two patient clusters were formed, based on the expression of the 6 genes belonging to Group 2. Interestingly, survival analysis on these patient clusters revealed that low expression of Group2 genes, is associated with poor prognosis (log rank *p*-value = .096; hazard ratio 0.7217, 95% confidence interval 0.4809 to 1.083) (Fig. 8). Moreover, combining the aforementioned information from both gene groups, 4 clusters of patients were formed. Pairwise survival analysis on all 4 clusters, showed that the 22 gene signature could define patient clusters with differences in overall survival estimates. In detail, the most significant differences, were identified for the two following comparisons: “HighGroup1.HighGroup2 vs. HighGroup1.LowGroup2” (log rank p-value = .037; hazard ratio 0.4736, 95% confidence interval 0.2303 to 0.9739), “HighGroup1.HighGroup2 vs. LowGroup1.LowGroup2” (log rank p-value = .087; hazard ratio 0.5526, 95% confidence interval 0.2724 to 1.121). Overall, Group2 low expression is correlated with unfavorable survival, whereas high expression of Group1 is associated with a favorable prognosis (Fig. 9). Interestingly, the vast majority of the 6 genes representing the second sub-group of the signature (*ENO1, GSTP1, MTHFD1* & *SCD*), are mainly implicated in biological processes related to metabolism, such as carboxylic acid metabolic process, oxoacid metabolic process and unsaturated fatty acid metabolic process. Furthermore, *CD44* and *GSTP1* genes, are also annotated to other distinct molecular mechanisms, including apoptosis and growth factor signaling cascades. Regarding the sub-Group1, the 16 related genes are associated to other distinct metabolic processes, including glycogen metabolic process, lipid metabolic process and bicarbonate transport.

## 4 Discussion

In this study, we propose a generic methodology for patient data integration (here, gene expression and PET-kinetic data) for tumor classification and survival analysis. We showcase the application of the methodology to CRC patients using two microarray datasets in combination with available PET data.

An initial important step was the extraction of gene expression signatures as putative biomarker sets with noted mechanistic, interpretation efficiency in terms of the molecular description of the pathology. These consist of pivotal genes in the cross-talk of systemic cellular processes [23]. Massive, existing functional and molecular pathway annotations, in the form of hierarchical vocabularies or ontologies, served as the drivers of the initial selection process, rather than solely quantitative, expression-based, computational techniques, engulfing the underlying molecular background. Classical, data mining methods for dimensionality reduction, when applied as the sole criterion for biomarker selection, without prior exploitation of recorded biological information, fail to capture molecular interactions and result to over-fitting, and loss of generalization power.

The initial gene signature of 94 DE attained efficient mapping onto significant processes of tumor biology and served as a basis for further derivation of compact signatures, including the final, composite one, integrating gene expression with molecular imaging data from PET. The selected genes possess strong regulatory role, involved in diverse, cross-talking processes with broader impact in cellular physiology. Τhe combination of genes and PET parameters achieved superior performance measures and improved the discrimination accuracy between cancer and control samples. The merit of the integrative strategy was further supported by the MFA results, confirming the informational complementarity of PET and gene expression measurements. Furthermore, the reduced compact composite set of 7 genes and 5 PET variables, serves as an initial pool of candidate biomarkers, for future independent studies, encompassing both layers of data.

The association of various PET variables to distinct biological pathways aligns well with findings from previous studies [24,25]. To assess the clinical significance of the correlation between PET and gene expression, a 22 gene set was derived and evaluated in the TCGA dataset. Interestingly, this analysis, based on an independent cohort, highlighted biologically meaningful groups of patients with notable differences in survival. For example, patients characterized with relatively low expression of Group2-genes, had the worst survival estimates. These findings further support the notion that specific PET variables encapsulate important molecular information, fitting the pathophysiological course of the disease.

The genes of the final signature represent the molecular counterparts of the PET-kinetic variables, are mostly related as expected, to distinct modules of metabolic reprogramming, which is considered as a hallmark of cancer progression [26]. In detail, it is well known that the balance between fatty acid synthesis and oxidation is deregulated in cancer, supporting cancer growth [27], whereas one‑carbon metabolism, is also altered during the initial steps of CRC manifestation, to enhance malignant transformation [28]. It has been also recently demonstrated that fatty acid beta oxidation genesis specifically disrupted in CRC [29]. Among the relevant to fatty acid beta oxidation genes, *ACADM* contributing to the signature as a major regulator, has been characterized as favorable prognostic gene in CRC [30]. In addition, several lines of evidence support the aberrant regulation of various bicarbonate transporters in oncogenesis, highlighting the importance of intracellular pH homeostasis in cancer cell survival [31]. On the other hand, except metabolism, the derived genes are also associated with distinct modes of tumor physiology in CRC, such as detoxification (*GSTP1*) [32], metastasis (*KIT*) [33], immunomodulation & stem cell renewal (*CD44*) [34] and ER stress (*CCT7*) [35]. Finally, the aforementioned distinct metabolic pathways could be targeted by novel contrast agents, enabling better screening of crucial metabolic components like carbohydrate metabolism and glycosylation, previously characterized as promoting metastasis and invasiveness in CRC cell lines [36].

## 5 Conclusions

Overall, this work represents a pioneering effort for multilayered, signature-oriented, analysis of CRC, in the context of radio-genomics, inferring a composite signature with promising results for robust epidemiological stratification. Further elucidation on the interrelationships between genes and PET variables hold the promise for development of non-invasive approaches, regarding the timely monitoring and the rational therapeutic administration in CRC. Finally, the composite signature should be tested in future external validation studies with larger sample sizes, initially regarding its discriminatory potential, as also for associations with distinct CRC subtypes and clinicopathological characteristics.

## Funding

This work was co-funded by the Operational Program “Competitiveness, Entrepreneurship and Innovation 2014-2020” (co-funded by the European Regional Development Fund) and managed by the General Secretariat of Research and Technology, Ministry Of Education, Research & Religious Affairs, under the project “Innovative Nanopharmaceuticals: Targeting Breast Cancer Stem Cells by a Novel Combination of Epigenetic and Anticancer Drugs with Gene Therapy (INNOCENT)” (7th Joint Translational Call - 2016, European Innovative Research and Technological Development Projects In Nanomedicine) of the ERA-NET EuroNanoMed II, and by the Helmholtz European Partnering Program for the cooperation between DKFZ and NHRF to build the Athens Comprehensive Cancer Center (ACCC).

## Conflict of Interest

The authors declare that there is no conflict of interest regarding the publication of this article.

## Ethical Approval

All procedures performed in studies involving human participants were in accordance with the ethical standards of the institutional and/or national research committee and with the 1964 Helsinki declaration and its later amendments or comparable ethical standards.

## Appendix A Supplementary data

Supplementary material 1 Image 1

Supplementary material 2 Image 2

Supplementary material 3 Image 3

Supplementary material 4 Image 4
