# Supplementary material for: Radiogenomic Analysis of F-18-Fluorodeoxyglucose Positron Emission Tomography and Gene Expression Data Elucidates the Epidemiological Complexity of Colorectal Cancer Landscape
Source: Comput Struct Biotechnol J. 2019 Jan 25;17:177–85. doi: 10.1016/j.csbj.2019.01.007 (PMC6374701; doi:10.1016/j.csbj.2019.01.007)
Supplement: Supplementary file 1 — Supplementary material 1 [file mmc1.docx]

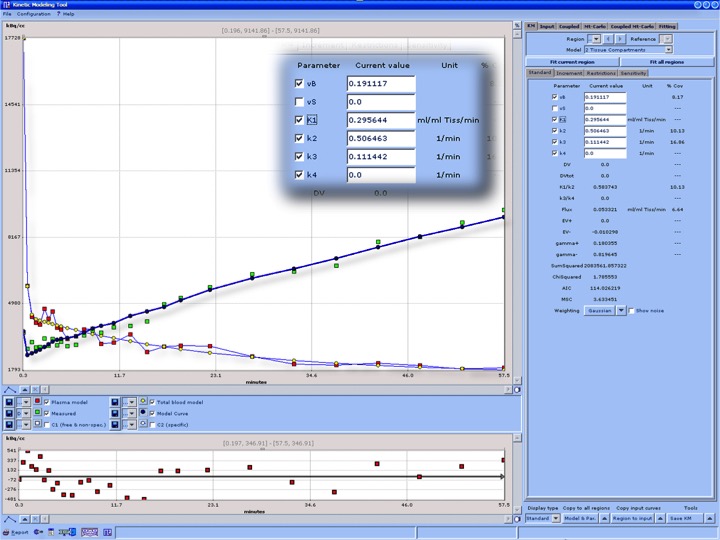


**Supplementary Figure S1.** Example of a PMOD Software evaluation for the extraction of the kinetic parameters. The curves demonstrate the time-activity data in the descending aorta, which serve as input data and in a tumor lesion. The descending aorta demonstrates a decreasing tracer concentration while the tumor shows a continuous increase up to 60 minutes post injection. The kinetic data are calculated based on a combination of a support vector machine algorithm and the Marquardt-Levenberg algorithm.


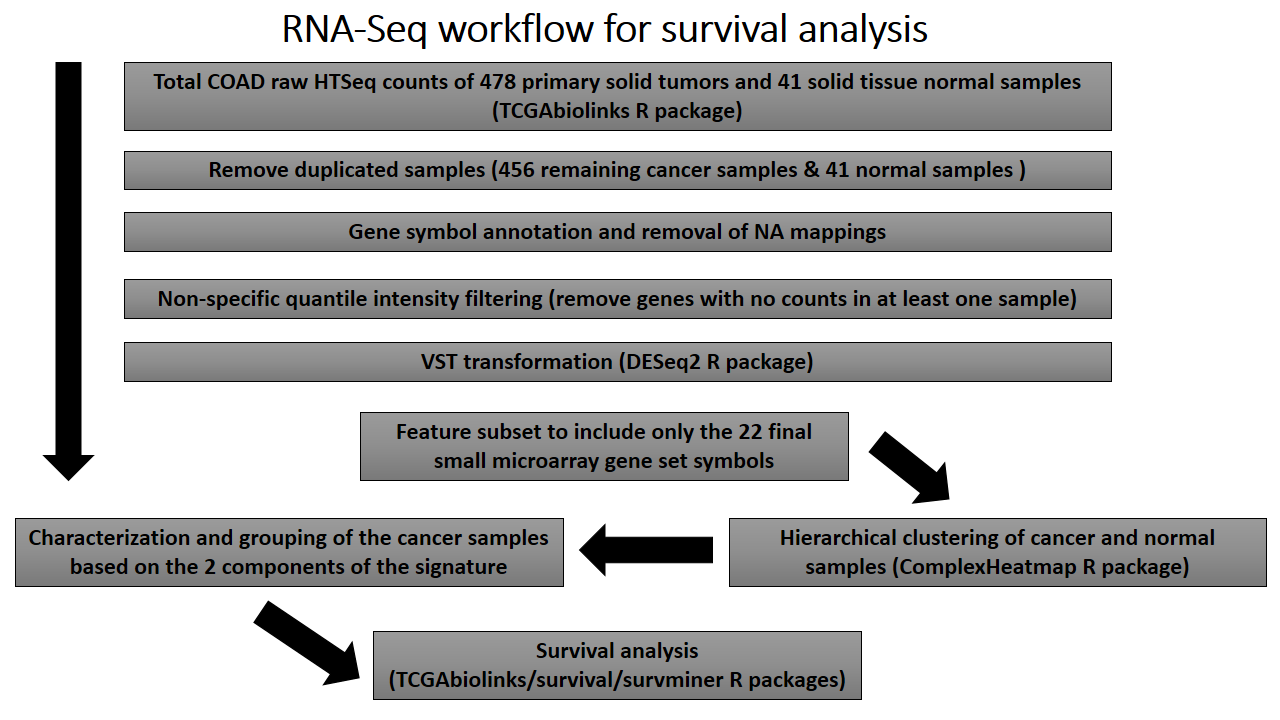


**Supplementary Figure S2.** The RNA-Seq workflow applied in the TCGA-COAD dataset with the molecular compact signature (22 selected genes): initially, for the hierarchical clustering both cancer and normal samples were included, whereas for the survival analysis only the cancer samples were utilized. Briefly, the grouping of the cancer samples was based on the 2 sub-groups of the 22-gene signature, resulting in 4 final patient clusters, which were used for evaluating any significant clinical impact in overall survival estimates (p-value of the log-rank test).

**Supplementary Figure S3.
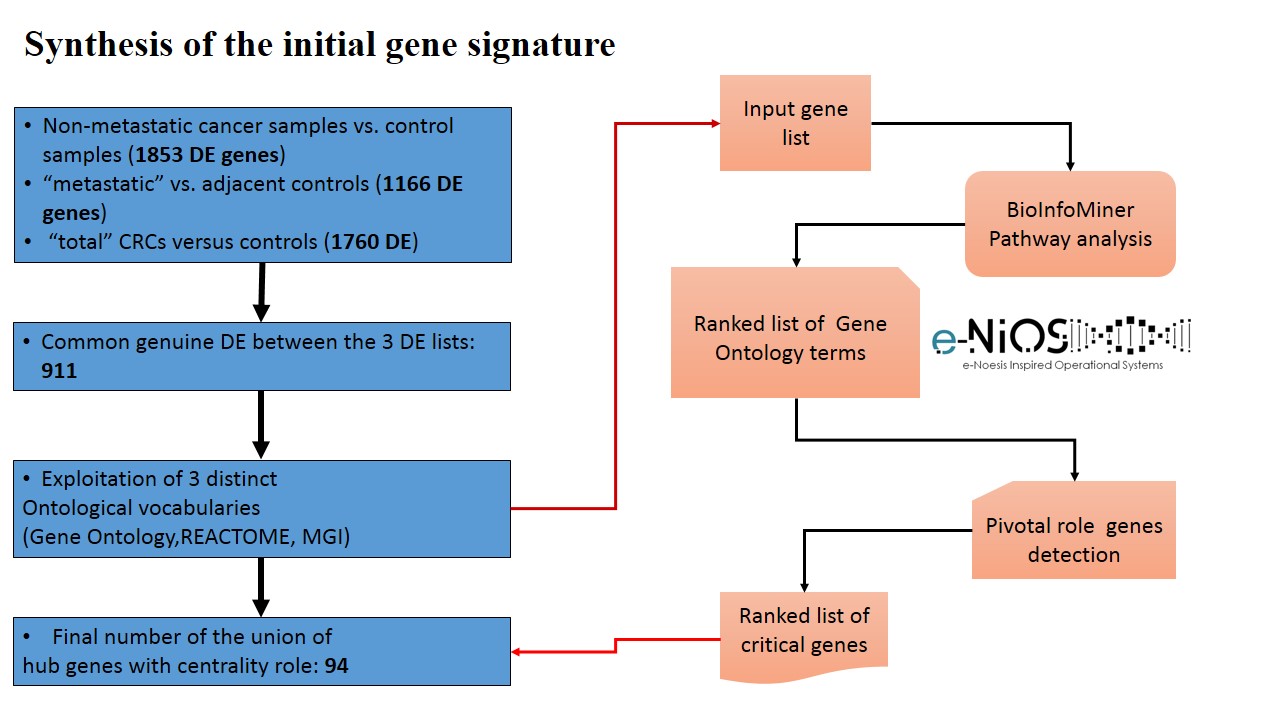
** Flowchart illustrating the development of the initial gene set, utilized for the derivation of the composite feature set. Briefly, this final subset resulted from the union of the three gene lists (94 genes in total), integrating the relevant information of the different layers of functional description, as performed by BioInfoMiner.
