# Supplementary material for: Radiogenomic Analysis of F-18-Fluorodeoxyglucose Positron Emission Tomography and Gene Expression Data Elucidates the Epidemiological Complexity of Colorectal Cancer Landscape
Source: Comput Struct Biotechnol J. 2019 Jan 25;17:177–85. doi: 10.1016/j.csbj.2019.01.007 (PMC6374701; doi:10.1016/j.csbj.2019.01.007)
Supplement: Supplementary file 2 — Supplementary material 2 [file mmc2.docx]

**Supplementary Legend of Figure 2**

1) The raw data for each dataset-cohort were imported and normalized separately with the R package frozen RMA (fRMA), which is a variant of Robust Multiarray Analysis algorithm (RMA) and flexibly allows to preprocess arrays in batches, and then combine the data for further analysis.

2) Then, the R package a4Base was used to merge the two datasets based on their common probesets (12142). Based on specific initial exploratory plots, we implemented the R package SVA and the ComBat method to perform batch effect correction, using as “batch” variable the “Study” variable (referring to each different cohort of patients).

3) We continued with a non-specific intensity filtering procedure, in order to remove probesets which are consistently non-expressed in most samples. To this extent, after the batch effect correction we removed the control probesets. Then, we plotted the density of the probe sets expression values -for each array separately- and used a metric (i.e. the highest peak of the distribution) as a value for an intensity based filter. Then, we removed all probe sets that had an expression value lower than the cut (in each array)-not expressed in more than 50% in either “CONTROL” or “CANCER” group samples, based on the phenotype variable “Disease”.

4) In order to assess differential expression, we used the limma R package and various linear models with an effect for patient (paired) and sample type (tumor/normal) that also included array weights, to accommodate low quality/discordant samples were fitted (Richie et al., 2006; Ritchie et al., 2015) & moderated t-statistics (Smyth, 2004) with adjusted p-values (Benjamin & Hochberg, 1995) were computed. For subsequent multifactor comparisons (which included for instance the Meta_factor variable indicating the presence of synchronous metastasis), we also used the function duplicateCorrelation, in order to handle the correlation between the related RNA samples/patients (Limma 9.7 users Guide). Finally, in all statistical comparisons the same double cutoff was used: an FDR value < 0.05 & an absolute value of log2-fold change > 0.5.

5) For the annotation process we used custom CDF packages from Brain Array, based on Entrez Genes (Version 19). Moreover, we utilized a custom python script to isolate from each hgu133plus2 CEL file, the corresponding A CEL signals (the exact same probes with the hgu133a chip) and the B CEL signals (hgu133b chip).

6) Finally, regarding the PET variables, a small non parametric missing value imputation was applied, using Random Forests (3 out of the 60 samples had NA values in 7 of the 8 PET features), using the R package missForest.
