# Supplementary material for: Radiogenomic Analysis of F-18-Fluorodeoxyglucose Positron Emission Tomography and Gene Expression Data Elucidates the Epidemiological Complexity of Colorectal Cancer Landscape
Source: Comput Struct Biotechnol J. 2019 Jan 25;17:177–85. doi: 10.1016/j.csbj.2019.01.007 (PMC6374701; doi:10.1016/j.csbj.2019.01.007)
Supplement: Supplementary file 3 — Supplementary material 3 [file mmc3.docx]

| Sample name | Anatomic location | Presence of synchronous distant metastasis | Tumor_Stage | Lymph_Node_Status | Sex | chip name or GEO platform id |
| --- | --- | --- | --- | --- | --- | --- |
| Patient 1 | coecum | No | T3 | N0 | F | HG-U133A |
| Patient 2 | rectosigmoid | No | T3 | N0 | M | HG-U133A |
| Patient 3 | c.ascending | No | T3 | N1 | F | HG-U133A |
| Patient 4 | c.ascending | No | T3 | N0 | F | HG-U133A |
| Patient 5 | right colic flexure | No | T2 | N0 | F | HG-U133A |
| Patient 6 | rectum | Yes | T3 | N2 | M | HG-U133A |
| Patient 7 | sigmoid | No | T3 | N1 | M | HG-U133A |
| Patient 8 | right colic flexure | No | T3 | N0 | M | HG-U133A |
| Patient 9 | coecum | Yes | T4 | N2 | F | HG-U133A |
| Patient 10 | right colic flexure | Yes | T4 | N2 | F | HG-U133A |
| Patient 11 | rectum | No | T3 | N0 | M | HG-U133A |
| Patient 12 | left colic flexure | No | T3 | N2 | M | HG-U133A |
| Patient 13 | sigmoid | Yes | T3 | N0 | F | HG-U133A |
| Patient 1 | coecum | Yes | T3 | N2 | M | HG-U133_Plus_2 |
| Patient 2 | descending colon | No | T3 | N1 | M | HG-U133_Plus_2 |
| Patient 3 | rectum | No | T2 | N0 | F | HG-U133_Plus_2 |
| Patient 4 | sigmoid | No | T3 | N0 | F | HG-U133_Plus_2 |
| Patient 5 | sigmoid | No | T2 | N2 | F | HG-U133_Plus_2 |
| Patient 6 | coecum | No | T3 | N0 | F | HG-U133_Plus_2 |
| Patient 7 | rectum | No | T1 | N0 | M | HG-U133_Plus_2 |
| Patient 8 | coecum | Yes | T3 | N2 | F | HG-U133_Plus_2 |
| Patient 9 | descending colon | No | T3 | N2 | F | HG-U133_Plus_2 |
| Patient 10 | asceding colon | No | T3 | N0 | F | HG-U133_Plus_2 |
| Patient 11 | descending colon | No | T3 | N0 | M | HG-U133_Plus_2 |
| Patient 12 | sigmoid | No | T3 | N0 | M | HG-U133_Plus_2 |
| Patient 13 | coecum | No | T2 | N0 | F | HG-U133_Plus_2 |
| Patient 14 | rectosigmoid | No | T3 | N0 | M | HG-U133_Plus_2 |
| Patient 15 | asceding colon | No | T2 | N0 | M | HG-U133_Plus_2 |
| Patient 16 | asceding colon | No | T2 | N0 | M | HG-U133_Plus_2 |
| Patient 17 | right colic flexure | No | T2 | N0 | M | HG-U133_Plus_2 |

**Supplementary Table S1.** Detailed clinicopathological characteristics of the 30 total patients in the two microarray datasets, including TNM staging, anatomic tumor location and sex.

|  | row | column | cor | p |
| --- | --- | --- | --- | --- |
| 1 | k3 | INF | 0.869575 | 0 |
| 2 | SUV | FD | 0.780661 | 1.91E-13 |
| 3 | SUV | k3 | 0.751487 | 4.58E-12 |
| 4 | FD | INF | 0.708252 | 2.46E-10 |
| 5 | k3 | FD | 0.689914 | 1.08E-09 |
| 6 | k1 | k2 | 0.674576 | 3.43E-09 |
| 7 | SUV | ACSF2 | -0.67108 | 4.42E-09 |
| 8 | INF | CCNJL | -0.66669 | 6.06E-09 |
| 9 | SUV | CA4 | -0.65718 | 1.18E-08 |
| 10 | SUV | PBLD | -0.6519 | 1.68E-08 |
| 11 | SUV | AQP8 | -0.64718 | 2.30E-08 |
| 12 | SUV | MS4A12 | -0.64568 | 2.54E-08 |
| 13 | SUV | CA1 | -0.64107 | 3.43E-08 |
| 14 | SUV | SLC26A2 | -0.6399 | 3.70E-08 |
| 15 | SUV | CHP2 | -0.63645 | 4.61E-08 |
| 16 | SUV | SNRPB | 0.631342 | 6.36E-08 |
| 17 | FD | SLC26A2 | -0.63018 | 6.84E-08 |
| 18 | SUV | PLCD1 | -0.62956 | 7.11E-08 |
| 19 | SUV | EXOSC2 | 0.627341 | 8.15E-08 |
| 20 | SUV | MARCH8 | -0.62723 | 8.21E-08 |
| 21 | SUV | EPHX2 | -0.6244 | 9.76E-08 |
| 22 | k3 | NMT2 | -0.62301 | 1.06E-07 |
| 23 | SUV | PPAP2A | -0.6229 | 1.07E-07 |
| 24 | SUV | FEZ2 | -0.62284 | 1.07E-07 |
| 25 | SUV | SLC22A5 | -0.62262 | 1.09E-07 |
| 26 | SUV | SLC26A3 | -0.62184 | 1.14E-07 |
| 27 | SUV | TDP2 | -0.62156 | 1.16E-07 |
| 28 | SUV | C1orf115 | -0.62101 | 1.20E-07 |
| 29 | SUV | WSCD1 | -0.62045 | 1.24E-07 |
| 30 | SUV | CALU | 0.619728 | 1.29E-07 |
| 31 | SUV | HSD17B11 | -0.61951 | 1.31E-07 |
| 32 | SUV | SLC25A20 | -0.61951 | 1.31E-07 |
| 33 | SUV | MSH6 | 0.617838 | 1.45E-07 |
| 34 | SUV | ACAT1 | -0.61712 | 1.51E-07 |
| 35 | SUV | NMT2 | -0.617 | 1.52E-07 |
| 36 | SUV | METTL7A | -0.616 | 1.61E-07 |
| 37 | SUV | SCD | 0.615282 | 1.68E-07 |
| 38 | SUV | G3BP1 | 0.615171 | 1.70E-07 |
| 39 | SUV | MTHFD1 | 0.614171 | 1.80E-07 |
| 40 | FD | ACSF2 | -0.6135 | 1.87E-07 |
| 41 | SUV | CLCA4 | -0.61323 | 1.90E-07 |
| 42 | SUV | S100A11 | 0.612337 | 2.00E-07 |
| 43 | SUV | CD44 | 0.612281 | 2.01E-07 |
| 44 | k3 | IGSF3 | -0.61223 | 2.01E-07 |
| 45 | SUV | PDGFD | -0.61084 | 2.18E-07 |
| 46 | SUV | PLSCR4 | -0.61006 | 2.29E-07 |
| 47 | SUV | ZG16 | -0.61 | 2.29E-07 |
| 48 | SUV | NT5DC2 | 0.607836 | 2.60E-07 |
| 49 | SUV | ARHGAP44 | -0.60784 | 2.60E-07 |
| 50 | SUV | TRANK1 | -0.60606 | 2.88E-07 |
| 51 | SUV | MXI1 | -0.60545 | 2.98E-07 |
| 52 | SUV | CDCA4 | 0.605279 | 3.01E-07 |
| 53 | SUV | STAB1 | -0.6035 | 3.33E-07 |
| 54 | SUV | ADAMDEC1 | -0.60283 | 3.45E-07 |
| 55 | SUV | PGM1 | -0.60245 | 3.53E-07 |
| 56 | SUV | ENO1 | 0.600889 | 3.85E-07 |
| 57 | k3 | CCNJL | -0.60028 | 3.99E-07 |

**Supplementary Table S6.** Correlation table of the significant correlation coefficients (Spearman correlation coefficient p.value < 0.05 and an absolute value >0.6) between the 8 PET variables and 911 DE genes, using the Hmisc R package.
